# Oxidation- and Temperature-Responsive Poly(hydroxyethyl acrylate-*co*-phenyl vinyl sulfide) Micelle as a Potential Anticancer Drug Carrier

**DOI:** 10.3390/pharmaceutics11090462

**Published:** 2019-09-06

**Authors:** Tae Hoon Kim, Madhusudhan Alle, Jin-Chul Kim

**Affiliations:** Department of Medical Biomaterials Engineering, College of Biomedical Science and Institute of Bioscience and Biotechnology, Kangwon National University, 192-1, Hyoja 2 dong, Chuncheon 200-701, Kangwon-do, Korea

**Keywords:** poly(hydroxyethyl acrylate-*co*-phenyl vinyl sulfide), oxidizable amphiphilic polymer, micelle, doxorubicin, oxidation- and temperature-responsive release, anticancer efficacy

## Abstract

Poly(hydroxyethyl acrylate-*co*-phenyl vinyl sulfide) (P(HEA-*co*-PVS)), as an oxidizable amphiphilic polymer, was prepared for the fabrication of an oxidation- and temperature-responsive micelle for the delivery of doxorubicin (DOX). The interfacial activity of H_2_O_2_-treated P(HEA-*co*-PVS) was significantly lower than that of the untreated variety, possibly because of the oxidization of PVS. P(HEA-*co*-PVS) exhibited a lower critical solution temperature (LCST) behavior and the LCST increased upon H_2_O_2_ treatment. The copolymer micelles, prepared by the dialysis method, were found to be round particles (less than 100 nm) on TEM micrograph. The release degree of Nile red loaded in the micelles was higher when the H_2_O_2_ concentration was higher, possibly because the micelles could be solubilized more readily at a higher H_2_O_2_ concentration. The release degree was more strongly dependent on the oxidizing agent concentration when the temperature was higher. DOX loaded in the micelles suppressed the in vitro growth of KB cells (a human cancer cell type originating from the cervix) much more effectively than DOX loaded in an unoxidizable control micelle and free DOX, possibly because the copolymer would undergo an increase in its LCST, lose its amphiphilic property, and the micelles would be disassembled. The DOX-loaded micelles were readily internalized into KB cells, as evidenced by flow cytometry (FACS) and confocal laser scanning microscopy (CLSM).

## 1. Introduction

Redox-responsive carriers have been of great interest in the field of drug delivery because they are able to release their payload specifically at the site of action where reducing agents and/or oxidizing one are relatively abundant. Various kinds of reduction-responsive drug carriers have been designed to deliver pharmaceutical agents intracellularly because the intracellular space, due to its higher concentration of glutathione, is more reductive than the extracellular space [1,2,3,4,5]. Since disulfide compounds can be readily reduced and broken down into thiol compounds, they have been exploited as major components for the preparation of reduction-responsive carriers [6,7,8,9,10]. Once the carriers are taken up by cells and reduced, their wall and/or matrix deteriorates, giving rise to promoted release. Nanogels, polymeric micelles, polymersomes, hyper-branched polymers, dendrons, layer-by-layer capsules, and cubic phases have been developed as reduction-responsive carriers, taking advantage of the reducible property of the disulfide bond [11,12,13,14,15,16].

On the other hand, oxidation-sensitive drug carriers were prepared to release pharmaceutical agents specifically at the site or in the condition of high reactive oxygen species (ROS) concentration. Since ROS can be created in high concentration in diseases such as rheumatoid arthritis, neurodegenerative diseases, atherosclerosis, diabetes, and cancers, oxidation-sensitive drug carriers can be utilized to release pharmaceutical agents specifically at the diseased sites or in the pathological condition [17,18]. Micelles were prepared using a diblock polymer of propylene sulfide and *N*,*N*-dimethylacrylamide for the specific release of drug at sites of high ROS activity (sites of inflammation) [19]. Upon oxidation, the copolymer lost its amphiphilicity because hydrophobic propylene sulfide could be converted to hydrophilic propylene sulfone. As a result, the micelles were dissembled and oxidation-triggered release took place. Oxidation-responsive polymeric vesicles were prepared using a poly(ethylene glycol)-poly(propylene sulfide)-poly(ethylene glycol) triblock copolymer as a building block [20]. When placed in an oxidative environment, the hydrophobic poly(propylene sulfide) block was converted to more hydrophilic poly(sulfones) and the triblock copolymer lost its amphiphilic property, leading to the disintegration of the vesicle. Oxidation-responsive microparticles were fabricated using arylboronic esters-modified dextran for the oxidation-triggered release of a model vaccine (i.e., ovalbumin) [21].

**Figure 1:**
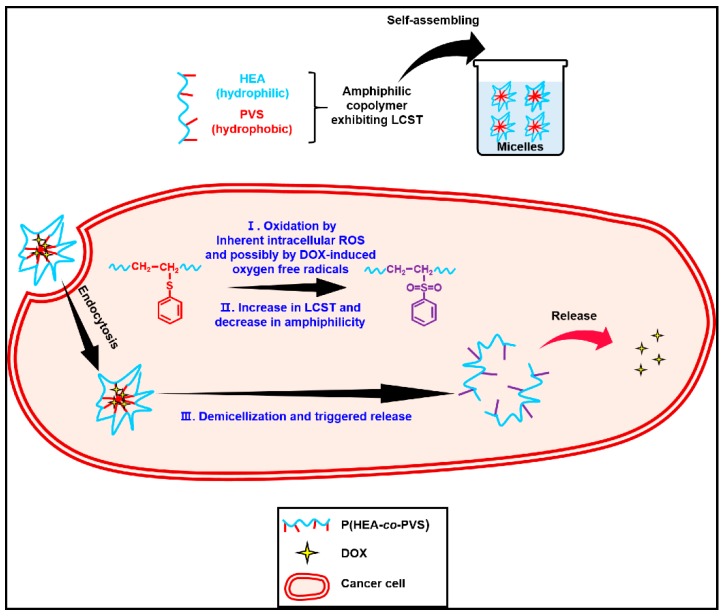
Schematic of oxidation- and temperature-responsive poly(hydroxyethyl acrylate-*co*-phenyl vinyl sulfide) P(HEA-*co*-PVS) micelle being used as a potential drug carrier for doxorubicin (DOX). Amphiphilic P(HEA-*co*-PVS) can be self-assembled into micelles in an aqueous solution. If the micelles are exposed to an oxidative environment, the PVS unit can be oxidized, the copolymer can undergo an increase in its lower critical solution temperature (LCST) and lose its amphiphilic property, and the micelles will be disassembled, resulting in an oxidation- and temperature-triggered release. Once the micelles are internalized into cancer cells, they can release their payload in response to the oxidative intracellular condition (inherent intracellular reactive oxygen species (ROS) plus DOX-induced oxygen free radicals), leading to enhanced anticancer efficacy.

In this study, an oxidation- and temperature-responsive polymeric micelle was prepared using poly(hydroxyethyl acrylate-*co*-phenyl vinyl sulfide) (P(HEA-*co*-PVS)) as an oxidizable amphiphilic lower critical solution temperature (LCST) copolymer. The copolymer is amphiphilic because the HEA segment is hydrophilic and PVS unit is hydrophobic; thus, it can be self-assembled into micelles in an aqueous solution. If the micelles are exposed to an oxidative environment, the PVS unit can be oxidized to more hydrophilic phenyl vinyl sulfoxide and/or sulfone; thus, the copolymer would undergo an increase in its LCST, lose its amphiphilic property, and the micelle would be disassembled, resulting in oxidation- and temperature-triggered release (Figure 1). Once the micelles are internalized into cancer cells, they would be subjected to oxidation due to the high intracellular ROS level. If doxorubicin (DOX, an anticancer agent) is loaded in the micelles, the intracellular oxidation of the micelles could be further expedited because DOX can form oxygen free radicals within cells. As a result, the oxidation- and temperature-responsive micelles would be able to release their payload in response to the oxidative intracellular condition (inherent intracellular ROS plus DOX-induced oxygen free radicals), leading to enhanced anticancer efficacy.

## 2. Materials and Methods

### 2.1. Materials

2-Hydroxyethyl acrylate (HEA, purity: 96%), *N,N*-dimethylformamide (DMF), phosphotungstic acid hydrate, dimethyl sulfoxide-d_6_ (DMSO-d_6_), thiazolyl blue tetrazolium bromide (MTT), fluoroshield^TM^ with 4’,6-diamidino-2-phenylindole (DAPI), triethylamine, and doxorubicin hydrochloride were purchased from Sigma Aldrich Chemical Co. (St. Louis, MO, USA). Phenyl vinyl sulfide (PVS), allylbenzene (ABZ), and Nile red were purchased from Tokyo Chemical Industry Co., Ltd. (Tokyo, Japan). Ethyl ether, hydrogen peroxide (H_2_O_2_), dimethyl sulfoxide (DMSO), and methanol were purchased from Daejung Chemicals & Metals Co., Ltd. (Siheung, South Korea). α,α’-Azobis(isobutyronitrile) (AIBN) was purchased from Junsei Chemical Co., Ltd. (Tokyo, Japan). 10X phosphate-buffered saline (PBS) was purchased from DYNEBIO (Seong-nam, South Korea). Roswell park memorial institute (RPMI) 1640 medium (no folic acid), phosphate-buffered saline (PBS), fetal bovine serum (FBS), trypsin-EDTA (0.25%), and penicillin‒streptomycin were purchased from Gibco^TM^ (Dublin, Ireland). KB cells were purchased from the Korean Cell Line Bank (Seoul, South Korea).

### 2.2. Preparation of Poly(hydroxyethyl acrylate-co-phenyl vinyl sulfide)

Poly(hydroxyethyl acrylate-*co*-phenyl vinyl sulfide) (P(HEA-*co*-PVS)) was prepared by free radical polymerization. HEA (4 g) and a variable amount of PVS (0 g, 0.047 g, 0.096 g, or 0.145 g) were dissolved together in 30 mL of DMF contained in a 100-mL three3-neck round-bottomed flask so that the HEA/PVS molar ratio was 100/0, 97/3, 98/2, or 99/1. The solution was purged with an N_2_ stream for 30 min, then heated to 73–75 °C by immersing the flask in a hot oil bath. AIBN (40 mg) was added to the heated solution and the reaction mixture was stirred for 12 h by a magnetic bar under an N_2_ atmosphere with reflux while being kept at the same temperature. After being cooled to a room temperature, the reaction mixture was poured in 500 mL of ethyl ether contained in a 1000-mL beaker to obtain the precipitate of the copolymer (P(HEA-*co*-PVS)). The precipitate, separated by filtration, was dissolved in 5 mL of DMF solution and re-precipitated in 500 mL of ethyl ether for the purification. The purified precipitate was filtered, washed with ethyl ether, and dried in a vacuum oven thermostated at 50 °C. As a control copolymer, poly(hydroxyethyl acrylate-*co*-allylbenzene) (P(HEA-*co*-ABZ)) was prepared by the same procedure described above, except that HEA (4 g) and ABZ (1.018 g) were co-dissolved in DMF so that the HEA/ABZ molar ratio was 80/20. The copolymer prepared using the reaction mixture whose HEA/PVS molar ratio was a/b was termed P(HEA-*co*-PVS)(a/b).

### 2.3. ^1^H NMR Spectroscopy

Dry copolymers (P(HEA-*co*-PVS) and P(HEA-*co*-ABZ)) were incubated with P_2_O_5_ in a vacuum oven thermostated at 50 °C in order to remove the bound solvent and water. A few milligrams of each of them were dissolved in DMSO-d_6_ and the copolymer solutions were subjected to ^1^H NMR spectroscopy on a NMR spectrophotometer (JNM-ECZ400S/L1 400 MHz, JEOL, Akishima, Japan). The acquisition time was 2.186 s, the sweep width was 7.494 kHz, and the recycle delay was 5.00 s.

### 2.4. Gel Permeation Chromatography (GPC)

The molecular weight of copolymers was determined by GPC (Breeze System, Waters, Milford, MA, USA). Copolymers were permeated through columns (Waters Ultrahydrogel Linear, 500, 250, and 120) and detected by a refractive index detector (Waters 2414). Pullulans (6100~642,000 gmol^−1^) were used as a standard polymer. The columns were eluted with NaNO_3_ solution (0.02 N) flowing at rate of 0.08 mL/min.

### 2.5. Examination of Oxidization of Sulfide Copolymer

One hundred milligrams each of P(HEA-*co*-PVS) and P(HEA-*co*-ABZ) copolymers was dissolved in 10 mL of H_2_O_2_ solution (1%, in water) contained in a 20-mL vial, tightly sealed, and left at room temperature for 6 h. The solutions were freeze-dried. P(HEA-*co*-PVS) and P(HEA-*co*-ABZ) treated with H_2_O_2_ solution were designated as H_2_O_2_-P(HEA-*co*-PVS) and H_2_O_2_-P(HEA-*co*-ABZ), respectively. The oxidation was confirmed by ^1^H NMR spectroscopy using the same operating condition and parameters described above.

### 2.6. Air/water Interfacial Tensiometry

The air/water interfacial tension of untreated and oxidized P(HEA-*co*-PVS) solutions was determined by a ring method. Fifteen milligrams of each of the copolymers were dissolved in PBS (10 mM. pH 7.4) so that the concentration was 1 mg/mL. Serial two-times dilution was done to obtain solutions of different concentrations. The air/water interfacial tension of the copolymer solutions was measured on a tensiometer (DST 60, SEO, Suwon-si, South Korea).

### 2.7. Observation of Temperature-Dependent Optical Density of Copolymer Solutions

Each of the H_2_O_2_-untreated copolymers and treated ones was dissolved in distilled water so that the concentration was 2% (*w/v*). Two and a half milliliters of each of the copolymer solutions were collected in a 3-mL glass cuvette and put in a cuvette hold equipped with a temperature controller (Peltier Controller, JENWAY, Staffordshire, UK). While being heated from 20 °C to 50 °C at a rate of about 2 °C /min, the optical density at 600 nm of the copolymer solutions was recorded on a UV spectrophotometer (6505 UV/VIS. Spectrophotometer, JENWAY, Staffordshire, UK).

### 2.8. Determination of Critical Micelle Concentration 

P(HEA-*co*-PVS)(97/3) and P(HEA-*co*-ABZ) were dissolved in distilled water so that the concentration was 0.0001‒1 mg/mL. Ten microliters of Nile red solution (1 mg/mL, in methanol) was added to 1 mL of copolymer solution in a 5-mL vial and rolled on a roller mixer overnight at room temperature under dark conditions. The fluorescence intensity of the solution was measured at 613 nm on a fluorescence spectrophotometer (Hitachi F2500, Hitachi, Tokyo, Japan) with excitation at 556 nm. The fluorescence intensity was plotted versus the logarithmic concentration of the copolymer solution.

### 2.9. Preparation of Micelles

Four milligrams of DOX-HCl and 1 μL of triethylamine were put in 2 mL of DMF contained in a 10-mL glass vial and it was rolled on a roller mixer overnight. In parallel, 1 mg of Nile red was dissolved in 10 mL of the same solvent. One hundred milligrams of a copolymer (i.e., P(HEA-*co*-PVS)(97/3) and P(HEA-*co*-ABZ)) were dissolved in 2 mL each of the DOX and Nile red solutions. The mixture was put in 20 mL distilled water (pre-adjusted to pH 7.4) contained in a 30-mL glass vial and stirred at room temperature for 4 h. The solutions were dialyzed against distilled water using a dialysis bag (MWCO 3500) for 48 h to obtain micellar solutions. The dialyzed solutions were freeze-dried for further use. Empty micelles were prepared by the same procedure described above, except DMF was used instead of the DOX and Nile red solutions.

### 2.10. Determination of Specific Loading of DOX in Micelles 

Ten milligrams of dry micelle were dissolved in 1 mL of DMF to determine the specific loading (%), defined as the percent of the mass of DOX loaded in a micelle based on the mass of the micelle. The DOX fluorescence intensity of the solution was measured at 550 nm on a fluorescence spectrophotometer (Hitachi F2500, Hitachi, Japan) with excitation at 485 nm. The amount of DOX corresponding to the fluorescence intensity was determined on a calibration curve.

### 2.11. Measurement of Hydrodynamic Diameter

Copolymer micelle solutions were diluted with distilled water so that the light-scattering intensity fell within 50‒200 kilo count per second on a dynamic light scattering instrument (Plus 90, Brookhaven Instruments, Holtsville, NY, USA). The hydrodynamic diameter was measured three times and the mean value was reported.

### 2.12. Transmission Electron Microscopy

Ten microliters each of copolymer micelle suspension and phosphotungstic acid solution (2%, in distilled water) were put in a 1.5-mL Eppendorf tube and they were mixed on a vortex mixer for a few seconds before they were stood at room temperature under dark conditions for 4 h. An aliquot of the mixture suspension was put on a formvar/copper-coated grid, any excess of the suspension was absorbed by filter paper, and the wet grid was air-dried at room temperature. The shape of the copolymer micelle was examined on a transmission electron microscope (JEM-2100F JEOL, Akishima, Japan).

### 2.13. Observation of Oxidation- and Temperature-Responsive Release 

First 0.4 mL of H_2_O_2_ solution (0%, 2.5%, or 5% in distilled water) was mixed with 1.6 mL of Nile red-loaded micelle solution contained in a 10-mL vial, and the mixture was gently stirred using a magnetic stirrer at room temperature under dark conditions. The fluorescence intensity of Nile red was measured at 637 nm on a fluorescence spectrophotometer (Hitachi F2500, Hitachi, Japan) with an excitation wavelength of 599 nm. Nile red exhibits its fluorescence in an apolar medium, but it hardly shows its fluorescence in a polar medium. Therefore, the fluorescence intensity is proportional to the amount of the dye loaded in the apolar core of the copolymer micelle and it decreases in proportion to the amount of the dye released from the apolar core to polar release medium (i.e., water). Thus, the release % of the dye can be determined using the following Equation (1) [22]:

(1) $$\mathrm{Release}\%=(1-(\frac{F_{t}}{F_{0}}))\times 100\%,$$

where *F*_0_ was the initial fluorescence intensity and *F_t_* was the fluorescence intensity at a given time.

### 2.14. Investigation of In Vitro Anticancer Efficacy 

P(HEA-*co*-PVS)(97/3)/DOX and P(HEA-*co*-ABZ)/DOX micelle solutions were diluted with PBS (10 mM, pH 7.4) so that the DOX concentration was 0.1, 1, 5, 10, 20, or 40 μg/mL. In parallel, empty micelle solutions were prepared as positive controls so that the copolymer concentrations were the same as those of the DOX-loaded micelle solutions. Free DOX solution in PBS (10 mM, pH 7.4) was prepared as another positive control so that the DOX concentration was the same as that of the DOX-loaded micelle solution. PBS (10 mM, pH 7.4) was used as a negative control. Then 200 μL of KB cell (1 × 10^4^ cells/mL) were seeded to each well of a 96-well plate and incubated for 24 h in a CO_2_ incubator at 37 °C (KB cell is a human cancer cell; its origin is the cervix, and it is utilized for anticancer efficacy testing of DOX [23]). The culture medium was discarded, 180 μL of FBS-free RPMI and 20 μL each of test samples were added to each well, and they were cultured for 24 h in a CO_2_ incubator at 37 °C. After the culture medium was discarded, the cancer cells were rinsed with PBS (10 mM, pH 7.4)), 20 μL of MTT reagent (5 mg/mL) was added to each well, and they were incubated for 4 h for the cellular conversion of MTT to formazan. The supernatant was removed from the wells and 200 μL of DMSO were put in each well to dissolve the formazan. The absorbance of the formazan solution was determined at 540 nm using a microplate reader (N10588, Thermo Fisher Scientific, Waltham, MA, USA). Cell viability was obtained by calculating the percent of the absorbance of formazan produced by cells treated with a test sample or a control, based on the absorbance of formazan produced by cells treated with the buffer solution. The cell growth inhibition was obtained using the cell viability; it was plotted versus DOX concentration, and the half maximal inhibitory concentration (IC_50_) of each sample was determined using the plot (i.e., cell growth inhibition versus DOX concentration). 

### 2.15. Observation of Cellular Internalization of DOX-Loaded Micelles 

One milliliter of KB cell suspension was seeded in a 12-well plate so that 3 × 10^4^ cells were included in each well; then they were incubated in a CO_2_ incubator at 37 °C for 24 h. After the culture medium was discarded, cells were rinsed with PBS (10 mM, pH 7.4) and 0.1 mL of RPMI 1640 culture medium free of FBS; the same amount of a test sample (free DOX solution, P(HEA-*co*-PVS)(97/3)/DOX micelle solution, and P(HEA-*co*-ABZ)/DOX micelle solution) was put in each well, and they were incubated for 0.5, 2, or 4 h. The culture medium was removed and the cells were rinsed with a buffer solution to remove free DOX and micelles. The cells were retrieved from the well wall using a trypsin/EDTA solution, put in an Eppendorf tube, centrifuged, and the supernatant decanted. The cells were re-suspended in 400 μL of PBS (10 mM, pH 7.4, 4 °C) and the fluorescence intensity of DOX was measured on a flow cytometry unit (FACS, FACS Calibur, Becton Dickinson, Franklin Lakes, NJ, USA). In parallel, confocal laser scanning microscopy (CLSM) was exploited to observe the interaction of free DOX or DOX-loaded micelles with the cells. The cell culture conditions were the same as used in the FACS study. Following the cells’ treatment with free DOX or DOX-loaded micelles, they were structurally fixed using 200 μL of formaldehyde solution (2.5% (*v/v*)). After the cells were rinsed with PBS (10 mM, pH 7.4), the nuclei were stained using DAPI, the free dye was washed out with the buffer solution, and fluorescence images were obtained on a CLSM (LSM 880 with Airyscan, Carl Zeiss, Oberkochen, Germany). 

## 3. Results and Discussion

### 3.1. ^1^H NMR Spectroscopy

**Figure 2:**
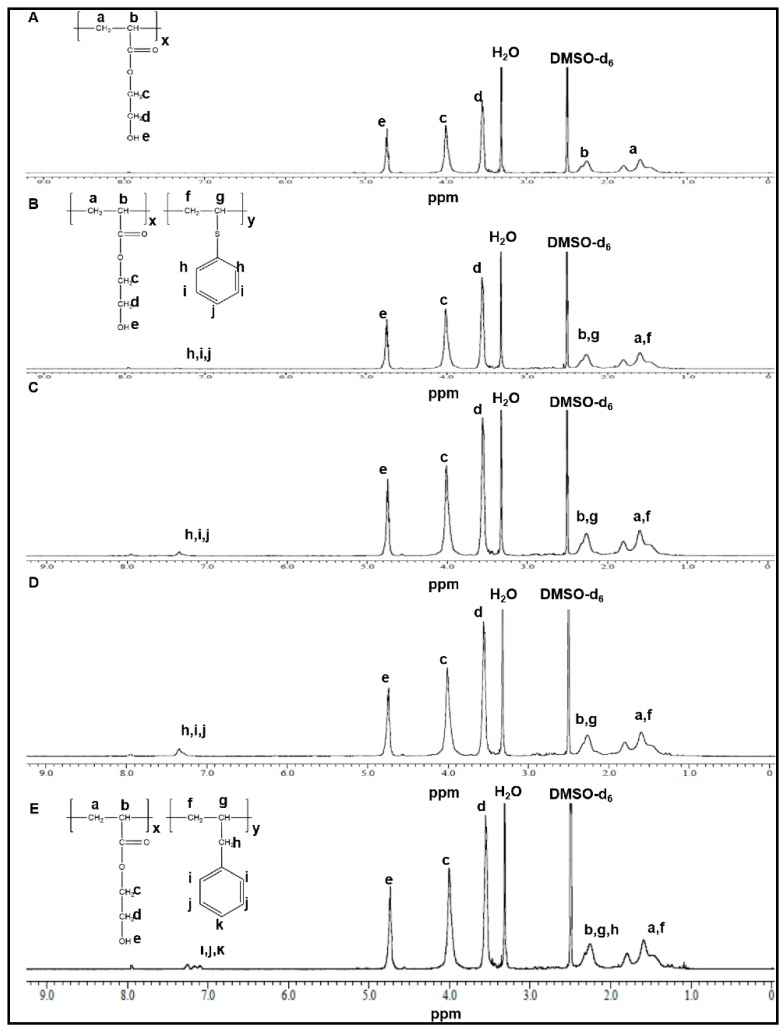
^1^H NMR spectrum of P(HEA-*co*-PVS)(100/0) (**A**), P(HEA-*co*-PVS)(99/1) (**B**), P(HEA-*co*-PVS)(98/2) (**C**), P(HEA-*co*-PVS)(97/3) (**D**), and P(HEA-*co*-ABZ) (**E**).

Figure 2A shows the ^1^H NMR spectrum of P(HEA-*co*-PVS)(100/0). The vinyl methylene group was found at 1.4–1.8 ppm, the vinyl methine group at 2.2–2.4 ppm, the methylene group next to the ester bond at 3.81–4.12 ppm, the methylene group adjacent to the hydroxyl group at 3.5–3.6 ppm, and the hydroxyl group at 4.7–4.8 ppm. Figure 2B shows the ^1^H NMR spectrum of P(HEA-*co*-PVS)(99/1). The vinyl methylene group of HEA was found at 1.35–1.85 ppm, the vinyl methine group of HEA at 2.2–2.4 ppm, the methylene group next to the ester bond at 3.8–4.2 ppm, the methylene group adjacent to hydroxyl group at 3.5–3.65 ppm, the hydroxyl group at 4.7–4.8 ppm, the vinyl methylene group of PVS at 1.35–1.85 ppm, the vinyl methine group of PVS at 2.2–2.4 ppm, and the phenyl group at 7.25–7.41 ppm. P(HEA-*co*-PVS)(98/2) and P(HEA-*co*-PVS)(97/3) exhibited their signals at the same position as P(HEA-*co*-PVS)(99/1) (Figure 2C,D). Using the signal area of the phenyl group and the hydroxyl group, the molar ratio of HEA/PVS of P(HEA-*co*-PVS)(99/1), P(HEA-*co*-PVS)(98/2), and P(HEA-*co*-PVS)(97/3) was calculated to be 99.5/0.5, 99.2/0.8, and 98.5/1.5, respectively. Figure 2E shows the ^1^H NMR spectrum of P(HEA-*co*-ABZ). The vinyl methylene group of HEA was found at 1.35–1.88 ppm, the vinyl methine group of HEA at 2.1–2.4 ppm, the methylene group next to the ester bond at 3.8–4.15 ppm, the methylene group adjacent to hydroxyl group at 3.41–3.62 ppm, the hydroxyl group at 4.68–4.82 ppm, the vinyl methylene group of ABZ at 1.35–1.88 ppm, the vinyl methine group of ABZ at 2.1–2.4 ppm, and the phenyl group at 7.05–7.3 ppm. Using the signal area of the phenyl group and the hydroxyl group, the molar ratio of HEA/ABZ of P(HEA-*co*-ABZ) was calculated to be 98.2/1.8.

### 3.2. GPC

Table 1 shows the molecular weight of copolymers determined by GPC. The number averaged molecular weight (M_n_) was 9764 to 10,522 and the polydispersity index (PDI) did not markedly deviate from 1 (1.17 to 1.20), indicating that the size distributions were quite narrow.

### 3.3. Examination of Oxidization of Sulfide Copolymers 

**Figure 3:**
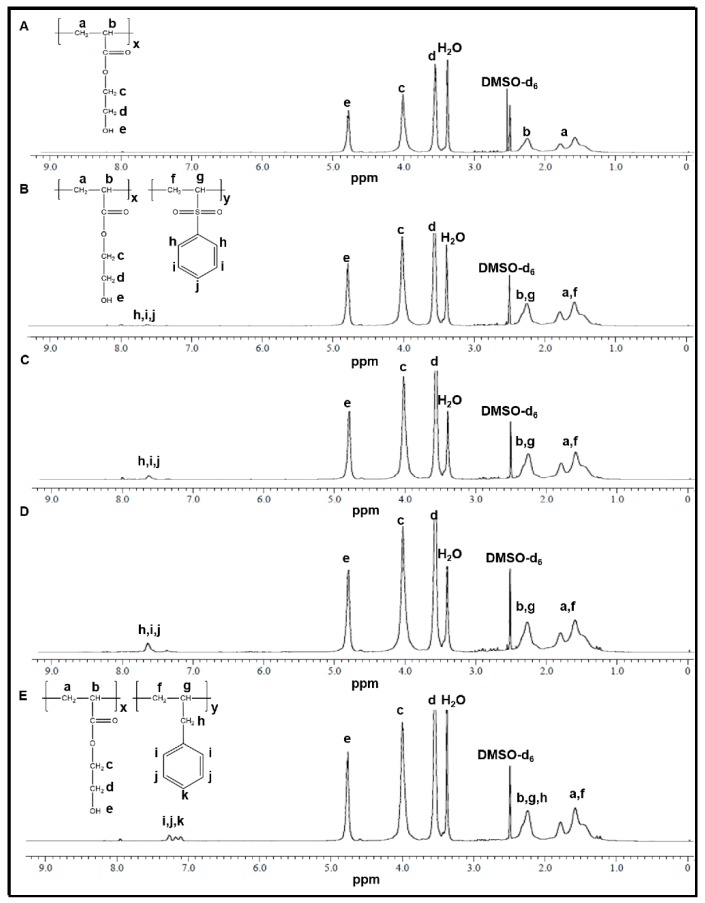
^1^H NMR spectrum of H_2_O_2_-P(HEA-*co*-PVS)(100/0) (**A**), H_2_O_2_-P(HEA-*co*-PVS)(99/1) (**B**), H_2_O_2_-P(HEA-*co*-PVS)(98/2) (**C**), H_2_O_2_-P(HEA-*co*-PVS)(97/3) (**D**), and H_2_O_2_-P(HEA-*co*-ABZ) (**E**).

H_2_O_2_-P(HEA-*co*-PVS)(100/0) showed their signals at the same position as P(HEA-*co*-PVS)(100/0) (Figure 3A). In fact, no oxidation-responsive groups were contained in the copolymer. H_2_O_2_-P(HEA-*co*-PVS)(99/1), H_2_O_2_-P(HEA-*co*-PVS)(98/2), and H_2_O_2_-P(HEA-*co*-PVS)(97/3) exhibited their signals at the same position as before treatment with H_2_O_2_, except that the signal of the phenyl group of PVS was shifted downfield by about 0.3 ppm (Figure 3B–D). This indicated that the sulfide was oxidized by H_2_O_2_ treatment. Meanwhile, the signal of the phenyl group of ABZ of P(HEA-*co*-ABZ) was not shifted after treatment with H_2_O_2_ (Figure 3E). There were no oxidizable groups in the copolymer.

### 3.4. Air/water Interfacial Tensiometry

**Figure 4:**
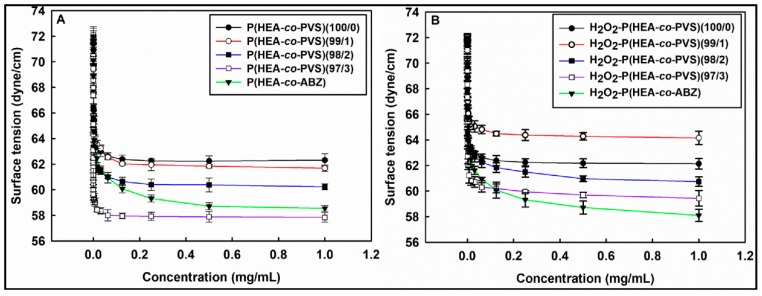
Air/water interfacial tension of copolymer (**A**) and H_2_O_2_-copolymer solutions (**B**).

Figure 4A shows the air/water interfacial tension of the P(HEA-*co*-PVS)(100/0), P(HEA-*co*-PVS)(99/1), P(HEA-*co*-PVS)(98/2), P(HEA-*co*-PVS)(97/3), and P(HEA-*co*-ABZ) solutions. The interfacial tension of the P(HEA-*co*-PVS)(100/0) solution steeply decreased from 72 dyne/cm to 62.3 dyne/cm in the concentration range of 0 to 1 mg/mL, and did not change significantly in the remaining concentration range. Since the vinyl backbone is hydrophobic and the pending group (i.e., hydroxyethyloxy carbonyl group) is relatively hydrophilic, P(HEA-*co*-PVS)(100/0) (i.e., homopolymer of HEA) is likely to be amphiphilic and interface-active. The HEA/PVS copolymer solutions exhibited interfacial tension profiles similar to that of the HEA homopolymer solution. However, the interfacial tension in the plateau region was lower as the PVS content was higher. For example, the interfacial tension at 1 mg/mL of the P(HEA-*co*-PVS)(100/0), P(HEA-*co*-PVS)(99/1), P(HEA-*co*-PVS)(98/2), and P(HEA-*co*-PVS)(97/3) solutions were 62.3 dyne/cm, 61.7 dyne/cm, 60.2 dyne/cm, and 57.8 dyne/cm, respectively. PVS would be able to increase the amphiphilicity and the interfacial activity of the polymeric chains if it was copolymerized with HEA, because it is a hydrophobic monomer. This would be a reason why the interfacial tension decreased when increasing the PVS content. On the other hand, the interfacial tension profile of the P(HEA-*co*-ABZ) solution resembled that of P(HEA-*co*-PVS)(97/3), and the plateau interfacial tension of the former was not significantly different from that of the latter. For example, the interfacial tension at 1 mg/mL of the P(HEA-*co*-ABZ) solution was 58.5 dyne/cm, almost the same as that of the P(HEA-*co*-PVS)(97/3) solution, at 57.8 dyne/cm. Figure 4B shows the air/water interfacial tension of the H_2_O_2_-P(HEA-*co*-PVS)(100/0), H_2_O_2_-P(HEA-*co*-PVS)(99/1), H_2_O_2_-P(HEA-*co*-PVS)(98/2), H_2_O_2_-P(HEA-*co*-PVS)(97/3), and H_2_O_2_-P(HEA-*co*-ABZ) solutions. The interfacial tensions decreased in the same fashion as the H_2_O_2_-untreated copolymer solutions. The interfacial tension profile of the H_2_O_2_-P(HEA-*co*-PVS)(100/0) solution was almost the same as that of the P(HEA-*co*-PVS)(100/0) solution, and the minimum interfacial tension value of the former solution, 62.1 dyne/cm, was not markedly different from that of the latter (62.3 dyne/cm). P(HEA-*co*-PVS)(100/0) (i.e., the HEA homopolymer) has no oxidizable groups in its structure, so it would be chemically stable in the H_2_O_2_ solution. On the other hand, the minimum interfacial tension value of the H_2_O_2_-P(HEA-*co*-PVS)(99/1) solution was 64.2 dyne/cm, significantly higher than that of the P(HEA-*co*-PVS)(99/1) solution (61.7 dyne/cm). The PVS of P(HEA-*co*-PVS)(99/1) would be oxidized to phenyl vinyl sulfone in the oxidative solution; it would become hydrophilic, and the amphiphilicity of the copolymer would be able to decrease, leading to a decrease in the interfacial activity. It is well known that sulfide is oxidized to sulfone under oxidative conditions [20]. The minimum interfacial tension values of the H_2_O_2_-P(HEA-*co*-PVS)(98/2) (60.7 dyne/cm) and H_2_O_2_-P(HEA-*co*-PVS)(97/3) solutions (59.4 dyne/cm) were also greater than those of the corresponding H_2_O_2_-untreated copolymer solutions. The oxidation of PVS was thought to be responsible for the decreased interfacial activity. After the copolymers were treated with H_2_O_2_ solution, the minimum interfacial tension value was greater, in the order of P(HEA-*co*-PVS)(99/1) solution > P(HEA-*co*-PVS)(98/2) solution > P(HEA-*co*-PVS)(97/3) solution, and the order was the same as before treatment with the oxidizing agent. This indicated that PVS was still relatively hydrophobic even after oxidization. Meanwhile, the interfacial tension profile of the H_2_O_2_-P(HEA-*co*-ABZ) solution was almost the same as that of the P(HEA-*co*-ABZ) solution, and the minimum interfacial tension value of the former solution, 58.1 dyne/cm, was close to that of the latter (58.5 dyne/cm). No oxidizable groups were contained in the structure of P(HEA-*co*-ABZ); thus, the chemical stability of the copolymer against H_2_O_2_ would be relatively high.

### 3.5. Observation of Temperature-Dependent Optical Density of Copolymer Solutions

**Figure 5:**
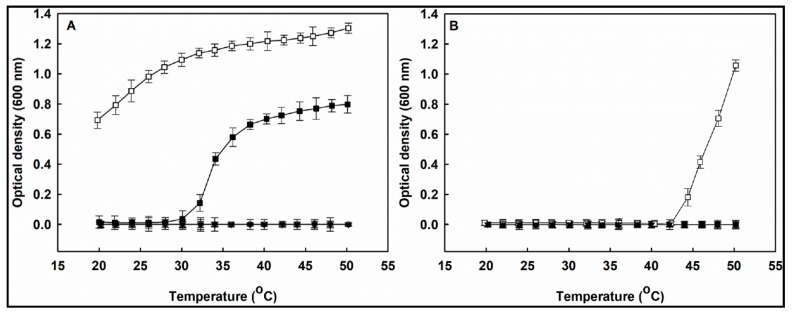
Optical density change of P(HEA-*co*-PVS)(100/0) (●), P(HEA-*co*-PVS)(99/1) (○), P(HEA-*co*-PVS)(98/2) (■), P(HEA-*co*-PVS)(97/3) (□), and P(HEA-*co*-ABZ) solutions (▼) with increasing temperature (**A**). Optical density change of H_2_O_2_-P(HEA-*co*-PVS)(100/0) (●), H_2_O_2_-P(HEA-*co*-PVS)(99/1) (○), H_2_O_2_-P(HEA-*co*-PVS)(98/2) (■), H_2_O_2_-P(HEA-*co*-PVS) (97/3) (□), and H_2_O_2_-P(HEA-*co*-ABZ) solutions (▼) with increasing temperature (**B**).

Figure 5A shows the optical density change of the P(HEA-*co*-PVS)(100/0), P(HEA-*co*-PVS)(99/1), P(HEA-*co*-PVS)(98/2), P(HEA-*co*-PVS)(97/3), and P(HEA-*co*-ABZ) solutions with increasing temperature. The optical density of the P(HEA-*co*-PVS)(100/0) and P(HEA-*co*-PVS)(99/1) solutions was almost zero in the full temperature range tested, indicating that the homopolymer and the copolymer were soluble in the temperature range. The optical density of the P(HEA-*co*-PVS)(98/2) solution was close to zero in the temperature range of 20 to 28 °C and thereafter steeply increased when increasing the temperature to reach 0.8 at 50 °C. This suggested that the copolymer was soluble in the lower temperature region and became insoluble when the temperature reached 30 °C. That is, the copolymer exhibited a lower critical solution temperature (LCST) behavior and the LCST was about 30 °C. If a hydrophobic monomer is copolymerized with HEA, a LCST copolymer can be obtained [24,25,26]. When the temperature of the medium is relatively low, HEA segments are hydrated, the hydrophobic interaction among PVS units can be suppressed, and the copolymer chains are likely to take an extended form. As the temperature increases, HEA segments become dehydrated, the hydrophobic interaction among PVS units is likely to become favorable, and the copolymer chains would be able to take a condensed form. This could account for why P(HEA-*co*-PVS)(98/2) showed LCST behavior. The optical density of the P(HEA-*co*-PVS)(97/3) solution was about 0.7 at 20 °C and gradually increased to 1.3 when the temperature increased to 50 °C. As in the case of P(HEA-*co*-PVS)(98/2), the temperature increase would lead to the dehydration of HEA segments of P(HEA-*co*-PVS)(97/3) and the hydrophobic interaction of PVS units of the copolymer, resulting in an optical density increase. Considering the trend of the optical density profile, the LCST of P(HEA-*co*-PVS)(97/3) seemed to be lower than 20 °C. The PVS content of P(HEA-*co*-PVS)(97/3) was higher than that of P(HEA-*co*-PVS)(98/2); thus, the thermally induced hydrophobic interaction of the hydrophobic unit (i.e., PVS) of the former copolymer would take place at a lower temperature. Since the LCST was higher when the PVS content was lower, the LCST of P(HEA-*co*-PVS)(99/1) might appear above 50 °C, if at all. On the other hand, the optical density of the P(HEA-*co*-ABZ) solution was almost zero in the full temperature range tested, suggesting that the copolymer was soluble and did not have LCST. 

Figure 5B shows the optical density change of the H_2_O_2_-P(HEA-*co*-PVS)(100/0), H_2_O_2_-P(HEA-*co*-PVS)(99/1), H_2_O_2_-P(HEA-*co*-PVS)(98/2), H_2_O_2_-P(HEA-*co*-PVS)(97/3), and H_2_O_2_-P(HEA-*co*-ABZ) solutions. The optical density of the H_2_O_2_-P(HEA-*co*-PVS)(100/0), H_2_O_2_-P(HEA-*co*-PVS)(99/1), and H_2_O_2_-P(HEA-*co*-PVS)(98/2) solutions was almost zero in the full temperature range tested. However, the optical density of the H_2_O_2_-P(HEA-*co*-PVS)(97/3) solution increased at around 44 °C (Figure 5B). Accordingly, it was concluded that the LCST of P(HEA-*co*-PVS)(98/2) disappeared after it was treated with H_2_O_2_. As described previously, the sulfide of PVS of P(HEA-*co*-PVS)(98/2) would be oxidized to sulfone by the oxidizing agent and the hydrophobicity of the PVS unit would decrease upon oxidation. Thus, the HEA segments need to be more dehydrated to thermally induce the hydrophobic interaction of oxidized PVS units and the thermal collapse of the copolymer chains. For more complete dehydration of the HEA segments, the copolymer solution need to be heated more. Thus, the LCST of P(HEA-*co*-PVS)(98/2) seemed to increase to over 50 °C after treatment with H_2_O_2_. This could explain why the LCST of P(HEA-*co*-PVS)(98/2) disappeared in the temperature window after the copolymer was treated with H_2_O_2_. The optical density of the H_2_O_2_-P(HEA-*co*-PVS)(97/3) solution was close to zero in the temperature range of 20–42 °C and thereafter steeply increased with the increase in temperature to reach almost 1.06 at 50 °C. That is, the LCST of H_2_O_2_-P(HEA-*co*-PVS)(97/3) was about 42 °C. The LCST of P(HEA-*co*-PVS)(97/3) was lower than 20 °C (Figure 5A). Thus, it could be said that the LCST of P(HEA-*co*-PVS)(97/3) increased after the copolymer was treated with H_2_O_2_. As described previously, the oxidation of PVS units could explain why the LCST of P(HEA-*co*-PVS)(97/3) increased after the copolymer was treated with H_2_O_2_. Meanwhile, the optical density of the H_2_O_2_-P(HEA-*co*-ABZ) solution was almost zero in the full temperature range tested. Considering that the optical density of the P(HEA-*co*-ABZ) solution was also almost zero in the same temperature range (Figure 5A), it seemed that the oxidizing agent had little effect on the phase transition of the copolymer in the temperature range tested. P(HEA-*co*-ABZ) had no oxidizable groups; thus, the copolymer would be chemically stable against H_2_O_2_.

### 3.6. Determination of Critical Micellization Concentration

**Figure 6:**
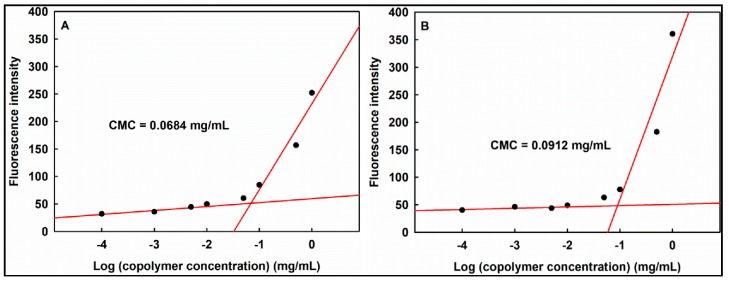
Nile red fluorescence intensity versus logarithmic copolymer concentration. Copolymer was P(HEA-*co*-PVS)(97/3) (**A**) and P(HEA-*co*-ABZ) (**B**).

Critical micellization concentration (CMC) corresponds to the intersection of two tangential lines on the plot of Nile red fluorescence intensity versus logarithmic copolymer concentration (Figure 6). Once the micelle is formed, Nile red can be dissolved into the micellar cores and emit stronger fluorescence [27,28,29]. The CMC of P(HEA-*co*-PVS)(97/3) and P(HEA-*co*-ABZ) were determined to be about 0.0684 and 0.0912 mg/mL, respectively. This suggested that the former copolymer would be micellized more readily than the latter. In fact, P(HEA-*co*-PVS)(97/3) was more interface-active than P(HEA-*co*-ABZ) (Figure 4A).

### 3.7. Determination of Specific Loading of DOX in Micelles 

The standard curve of DOX in DMF was expressed by an equation, Y = 1116.3 X + 49.6 (*R*^2^ = 0.9997), where X was the concentration of the DOX solution in μg/mL and Y was the fluorescence intensity of DOX. The specific loading of DOX in the P(HEA-*co*-PVS)(97/3) and P(HEA-*co*-ABZ) micelles was 0.16% and 0.17%, respectively. The HEA/PVS molar ratio of P(HEA-*co*-PVS)(97/3) was 98.5/1.5 and the HEA/ABZ molar ratio of P(HEA-*co*-ABZ) was 98.2/1.8. The hydrophobic unit content of the former copolymer was lower than that of the latter. Thus, if the mass of one particle of P(HEA-*co*-PVS)(97/3) micelle was the same as that of the P(HEA-*co*-ABZ) micelle, the hydrophobic domain of the former micelle would be smaller than that of the latter, accounting for the difference in the specific loading.

### 3.8. Measurement of Hydrodynamic Diameter

The mean hydrodynamic diameter of the P(HEA-*co*-PVS)(97/3) and P(HEA-*co*-ABZ) micelles was 94 nm and 88 nm, respectively. The HEA/PVS molar ratio of P(HEA-*co*-PVS)(97/3) was 98.5/1.5 and the HEA/ABZ molar ratio of P(HEA-*co*-ABZ) was 98.2/1.8. The hydrophobic unit content of the former copolymer was lower than that of the latter. Accordingly, the HEA segment of the former copolymer would be longer in its length than that of the latter. Thus, the hydrophilic corona of the P(HEA-*co*-PVS)(97/3) micelle would be able to extend more than that of the P(HEA-*co*-ABZ) micelle. This would be a reason why the mean diameter of the P(HEA-*co*-PVS)(97/3) micelle was somewhat larger than that of the P(HEA-*co*-ABZ) micelle. The mean hydrodynamic diameter of the P(HEA-*co*-PVS)(97/3)/DOX and P(HEA-*co*-ABZ)/DOX micelles was 98 nm and 91 nm, respectively. Since the diameters did not markedly deviate from those of empty micelles, it could be said that the loading of DOX had little effect on the diameter. DOX would be solubilized in the lipidic core of the micelle, which might affect the core size. However, it would hardly have an effect on the hydrodynamic diameter of the micelle because the mass of HEA segments was more than 98%; thus, the corona volume of the micelle would overwhelm the core volume.

### 3.9. Transmission Electron Microscopy (TEM)

**Figure 7:**
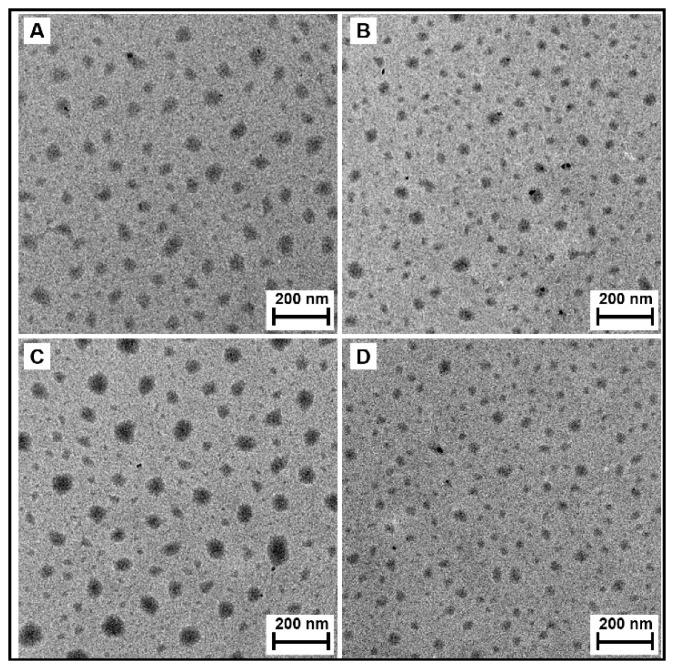
Transmission electron micrograph of P(HEA-*co*-PVS)(97/3) micelle (**A**), P(HEA-*co*-ABZ) micelle (**B**), P(HEA-*co*-PVS)(97/3)/DOX micelle (**C**), and P(HEA-*co*-ABZ)/DOX micelle (**D**). Bar on each micrograph represents 200 nm.

Figure 7A,B shows the TEM micrographs of the P(HEA-*co*-PVS)(97/3) and P(HEA-*co*-ABZ) micelles. The micelles were found to be round particles. No marked difference in shape and size was found between the micelles. The interfacial tension of the P(HEA-*co*-PVS)(97/3) solution was not markedly different from that of the P(HEA-*co*-ABZ) solution in the full range of concentration tested (Figure 4A), indicating that the interfacial activity of the former copolymer did not outstandingly deviate from that of the latter. In addition, the hydrophobic unit (i.e., PVS) of P(HEA-*co*-PVS)(97/3) was similar in chemical structure to that (i.e., ABZ) of P(HEA-*co*-ABZ). In this circumstance, the copolymers could be self-assembled without causing a big difference in the shape and size of the resulting micelles. The P(HEA-*co*-PVS)(97/3)/DOX and P(HEA-*co*-ABZ)/DOX micelles were also found to be round particles (Figure 7C,D), and were almost the same as the empty micelles in terms of shape and size.

### 3.10. Observation of Oxidation- and Temperature-Responsive Release

**Figure 8:**
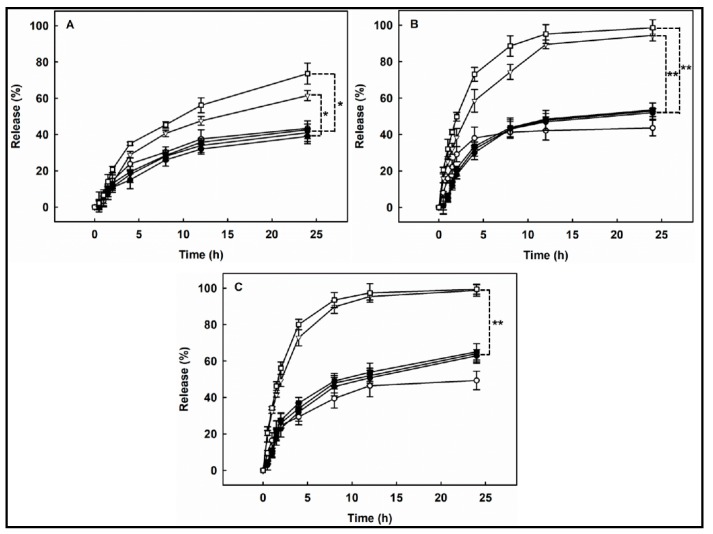
Release profile of Nile red loaded in P(HEA-*co*-ABZ) (filled symbols) and P(HEA-*co*-PVS)(97/3) micelles (empty symbols) at 25 °C (**A**), 37 °C (**B**), and 45 °C (**C**), when the H_2_O_2_ concentration of release medium was 0% (●, ○), 0.5% (▼, ▽), and 1.0% (■, □). The data are expressed as mean ± SD (*n* = 3) (* *p* < 0.01, ** *p* < 0.001 vs. the P(HEA-*co*-ABZ) micelle for each H_2_O_2_ concentration).

Figure 8 shows the release profile of Nile red loaded in P(HEA-*co*-ABZ) and P(HEA-*co*-PVS)(97/3) micelles at 25 °C, 37 °C, and 45 °C, when the H_2_O_2_ concentration of release medium was 0%, 0.5%, and 1.0%. At 25 °C, the release degree of Nile red loaded in the P(HEA-*co*-ABZ) micelle increased along a saturation curve for 24 h and was not markedly different regardless of the oxidizing agent concentration. For example, the release degree in 24 h was 39%, 41%, and 42.6%, respectively, when the concentration was 0%, 0.5%, and 1.0%. In fact, P(HEA-*co*-ABZ) had no oxidation-susceptible moiety. Thus, the P(HEA-*co*-ABZ) micelle would be stable against the oxidizing agent in terms of its integrity. As in the case of 25 °C, the release degree at 37 °C and 45 °C also increased along a saturation curve and was not markedly affected by the H_2_O_2_ concentration either. The release degree was higher in the order of 45 °C > 37 °C > 25 °C, possibly because of the thermally increased diffusivity of a diffusate and the thermally increased fluidity of the micellar core. Like the P(HEA-*co*-ABZ) micelle, the P(HEA-*co*-PVS)(97/3) micelle released its payload in a saturation manner at all the temperatures and H_2_O_2_ concentrations tested. At 25 °C, the release degree of Nile red loaded in the P(HEA-*co*-PVS)(97/3) micelle was higher as the H_2_O_2_ concentration was raised (Figure 8A). When no H_2_O_2_ was contained in the release medium, the release degree of Nile red loaded in the P(HEA-*co*-PVS)(97/3) micelle was insignificantly different from that of Nile red loaded in the P(HEA-*co*-ABZ) micelle. However, when the H_2_O_2_ concentration was 0.5% or 1.0%, the release degree of Nile red loaded in the P(HEA-*co*-PVS)(97/3) micelle was significantly higher than that of Nile red loaded in the P(HEA-*co*-ABZ) micelle. For example, the release degree in 24 h was about 43.4%, 61.5%, and 73.6%, respectively, when the H_2_O_2_ concentration was 0%, 0.5%, and 1.0%. The optical density of the P(HEA-*co*-PVS)(97/3) solution markedly decreased after oxidation at all the temperatures (25 °C, 37 °C, and 45 °C) adopted for the release experiment (Figure 5), suggesting that the solubility of the copolymer increased after the oxidation at those temperatures. Accordingly, if they came in contact with the oxidizing agent solution, the copolymer chains constituting the oxidizable micelle would be solubilized and the release degree would be increased. At 37 °C, the release degree of Nile red loaded in the P(HEA-*co*-PVS)(97/3) micelle was more strongly dependent on H_2_O_2_ concentration (Figure 8B). The release degree in 24 h was about 43.6%, 94.4%, and 98.5%, respectively, when the H_2_O_2_ concentration was 0%, 0.5%, and 1.0%. The release degree with H_2_O_2_ (0.5% and 1.0%) was about two times higher than the release degree of dye loaded in the P(HEA-*co*-ABZ) micelle at the same H_2_O_2_ concentration. Considering the optical density of the P(HEA-*co*-PVS)(97/3) solution at 37 °C (about 1.19) was higher than the optical density observed at 25 °C (about 0.93) (Figure 5A), the micelles at the higher temperature would have a stronger driving force for the oxidation-induced dissolution. At 45 °C, the release was sensitive to the oxidizing agent as much as the release at 37 °C (Figure 8C). The release degree in 24 h was about 49.4%, 98.7%, and 99.4%, respectively, when the H_2_O_2_ concentration was 0%, 0.5%, and 1.0%. The release degree with H_2_O_2_ (0.5% and 1.0%) was much higher than the release degree of dye loaded in the P(HEA-*co*-ABZ) micelle at the same H_2_O_2_ concentration. The P(HEA-*co*-PVS)(97/3) solution exhibited a high optical density at 45 °C (about 1.24) (Figure 5A), as it did at 37 °C.

### 3.11. Investigation of In Vitro Anticancer Efficacy

**Figure 9:**
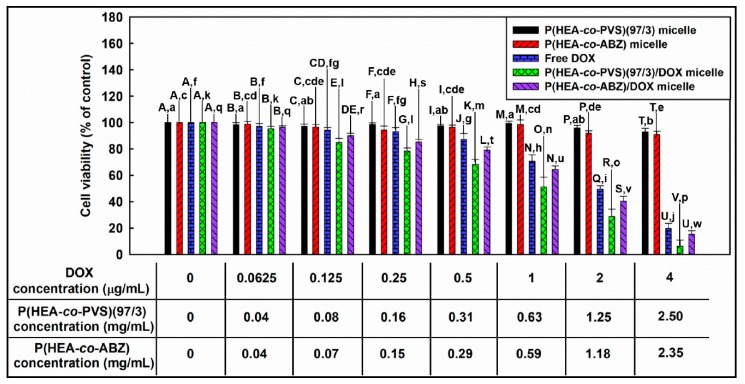
Viability of KB cells. Uppercase letters mean statistically significant differences in cell viability between the different samples when the doxorubicin concentration was the same. Lowercase letters represent statistically significant differences in cell viability between the same samples when the concentration of doxorubicin was different.

Figure 9 shows the viability of KB cells treated with P(HEA-*co*-PVS)(97/3) micelles, P(HEA-*co*-ABZ) micelles, free DOX, P(HEA-*co*-PVS)(97/3)/DOX micelles, and P(HEA-*co*-ABZ)/DOX micelles. The viability of cells treated with P(HEA-*co*-PVS)(97/3) micelles was almost 100% in the copolymer concentration range of 0.04 to 0.63 mg/mL and slightly decreased to 93% when the concentration increased to 2.50 mg/mL. Similarly, the viability of cells treated with P(HEA-*co*-ABZ) micelles did not decrease appreciably in the 0.04 to 0.59 mg/mL range, and fell to 91% when the concentration increased to 2.35 mg/mL. Thus, it could be said that the empty micelles exhibited no severe in vitro toxicity in the full concentration tested. The viability of cells treated with free DOX slightly decreased to about 87% in 0–0.5 μg/mL and markedly decreased to 22% when the concentration increased to 4 μg/mL. The viability of cells treated with P(HEA-*co*-PVS)(97/3)/DOX micelles decreased with DOX concentration in a similar manner; it was significantly lower than that of cells treated with free DOX at all the concentrations tested, except for the lowest concentration (i.e., 0.0625 μg/mL), and close to 0 at 4 μg/mL. Particular matter including polymeric micelles can be taken up by cancer cells via endocytosis [30]. Once the micelles are internalized into the cells, they can readily release their payload because the inside of the cancer cell is known to be oxidative due to a high ROS level [31,32]. In fact, P(HEA-*co*-PVS)(97/3) micelles exhibited a promoted release at 37 °C in an oxidative condition (Figure 8B). The viability of cells treated with P(HEA-*co*-ABZ)/DOX micelles decreased with DOX concentration in a similar fashion. In the full concentration range tested, the viability was not significantly lower than that of cells treated with free DOX, and was significantly higher than that of cells treated with P(HEA-*co*-PVS)(97/3)/DOX micelles. That is, despite P(HEA-*co*-ABZ))/DOX micelles also being a particulate carrier able to be internalized into the cancer cell, they were not as effective at suppressing cancer cell growth as P(HEA-*co*-PVS)(97/3)/DOX micelles. In fact, in an oxidative condition at 37 °C, P(HEA-*co*-ABZ) micelles could hardly promote the release of their payload, and thus released much less than the oxidation- and temperature-responsive micelles (i.e., P(HEA-*co*-PVS)(97/3) micelles) (Figure 8B). As shown in Table 2, the IC_50_ value of free DOX, P(HEA-*co*-PVS(97/3)DOX micelles, and P(HEA-*co*-ABZ)/DOX micelles were estimated to be 2.3, 1.62, and 2.02, respectively, suggesting that the anticancer efficacy was in the order of P(HEA-*co*-PVS(97/3)DOX micelles > P(HEA-*co*-ABZ)/DOX micelle > free DOX. 

### 3.12. Observation of Cellular Internalization of DOX-Loaded Micelles

**Figure 10:**
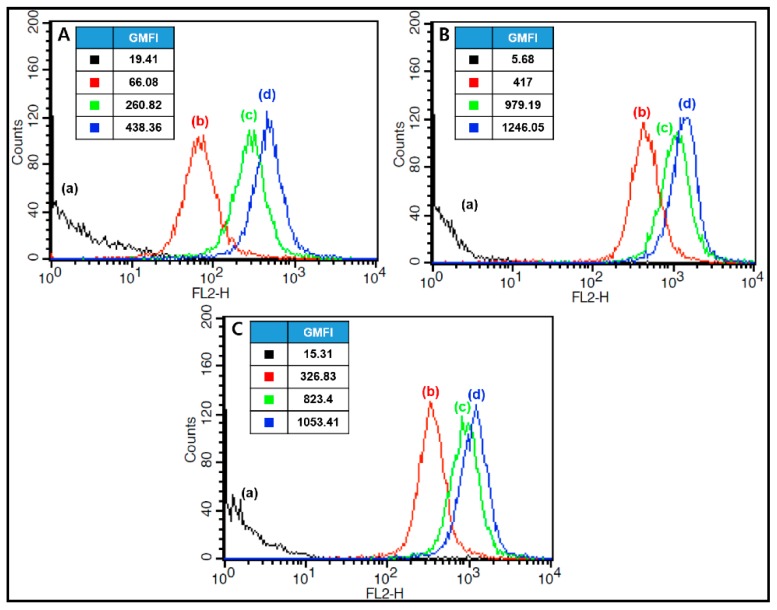
Flow cytometric profile of KB cells treated with free DOX (**A**), P(HEA-*co*-PVS)(97/3)/DOX micelles (**B**), and P(HEA-*co*-ABZ)/DOX micelles (**C**) after the treated cells were incubated for 0 (**a**), 0.5 (**b**), 2 (**c**) and 4 h (**d**).

Figure 10 shows the flow cytometric profile of KB cells treated with free DOX, P(HEA-*co*-PVS)(97/3)/DOX micelles, and P(HEA-*co*-ABZ)/DOX micelles after the treated cells were incubated for 0, 0.5, 2, or 4 h. The fluorescence intensity of cells treated with free DOX increased with the incubation period (Figure 10A). For example, the geometric mean fluorescence intensity (GMFI) was 66.08, 260.82, and 438.36, respectively, when the incubation period was 0.5 h, 2 h, and 4 h. DOX would be able to diffuse into the intracellular space owing to the concentration gradient across the cellular membrane. The GMFI of cells treated with P(HEA-*co*-PVS)(97/3)/DOX micelles was 417, 979.19, and 1246.05, respectively, when the incubation period was 0.5 h, 2 h, and 4 h (Figure 10B). The GMFI was stronger than that of cells treated with free DOX, implying that the micellar DOX was internalized more readily into cells than free DOX. This could account for why DOX loaded in P(HEA-*co*-PVS)(97/3) micelles exhibited higher anticancer efficacy than free DOX (Figure 9). Particulate carriers including micelles were reported to be taken up by cancer cells through phagocytosis [33,34]. The stronger GMFI could be ascribed to the endocytosis of the micelles into the cells. The GMFI of cells treated with P(HEA-*co*-ABZ)/DOX micelles was close to that of cells treated with P(HEA-*co*-PVS)(97/3)/DOX micelles at all the incubation times tested (Figure 10C). This suggested that DOX loaded in P(HEA-*co*-ABZ) micelles was internalized into the intracellular space as much as DOX loaded in P(HEA-*co*-PVS)(97/3) and was internalized more easily than free DOX. Nevertheless, DOX loaded in P(HEA-*co*-ABZ) micelles exhibited lower anticancer efficacy than DOX loaded in P(HEA-*co*-PVS)(97/3) micelles and did not show significantly higher anticancer efficacy than free DOX (Figure 9). In oxidative conditions, P(HEA-*co*-ABZ) was chemically stable (Figure 3E); the copolymer micelle could hardly promote the release of its payload, and it could not release as much as P(HEA-*co*-PVS)(97/3) micelles (Figure 8).

**Figure 11:**
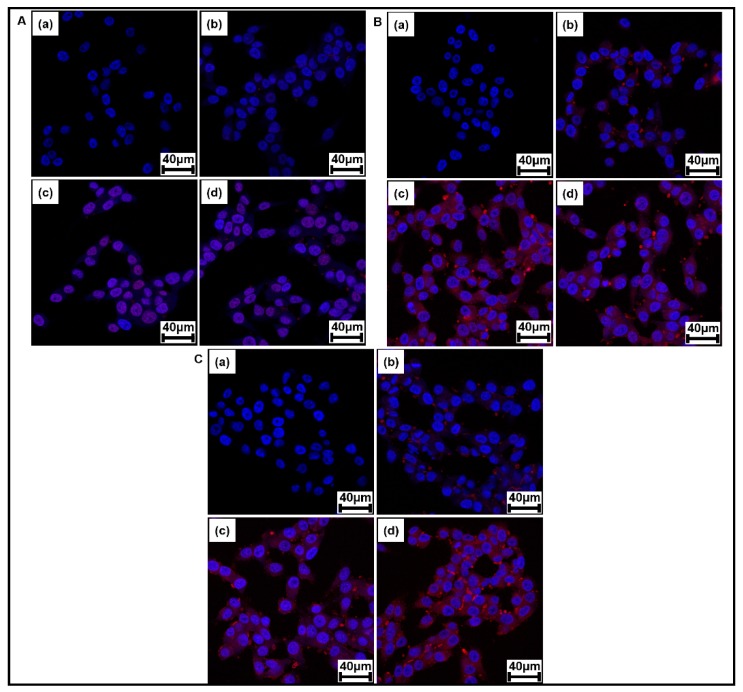
CLSM images of KB cells treated with free DOX (**A**), P(HEA-*co*-PVS)(97/3)/DOX micelles (**B**), and P(HEA-*co*-ABZ)/DOX micelles (**C**) after the treated cells were incubated for 0 (**a**), 0.5 (**b**), 2 (**c**) and 4 h (**d**).

Figure 11 shows the CLSM images of KB cells treated with free DOX, P(HEA-*co*-PVS)(97/3)/DOX micelles, and P(HEA-*co*-ABZ)/DOX micelles after the treated cells were incubated for 0, 0.5, 2, and 4 h. On the CLSM images of KB cells treated with free DOX, the DAPI-stained nuclei were seen as blue circular objects and the DOX fluorescence (red) appeared on and around the nuclei, indicating that DOX was internalized into the cells. The red color became more prominent over, but it was blurred throughout the incubation period. The DOX fluorescence of cells treated with P(HEA-*co*-PVS)(97/3)/DOX also appeared on and around the nuclei and became thicker with an increase in the incubation time. The DOX fluorescence was much stronger than that of cells treated with free DOX, possibly because of the endocytosis of the micelles into the cells. The DOX fluorescence of cells treated with P(HEA-*co*-ABZ)/DOX micelles was as intensive as that of cells treated with P(HEA-*co*-PVS)(97/3)/DOX micelles. The results of CLSM were in good agreement with those of FACS. Since the level of intracellular ROS might not be enough to oxidize P(HEA-*co*-PVS)(97/3), it was questionable whether the copolymer micelle could be oxidized and promote the release of its payload in the intracellular space. DOX is known to suppress the growth of cancer cells by intercalating between DNA double strands and inhibiting the biosynthesis of proteins. In addition, DOX was also reported to form oxygen free radicals and cause the peroxidation of cellular membrane lipids, leading to cancer cell death [35,36]. Besides inherent intracellular ROS, the oxygen free radicals produced by DOX would also help the copolymer to be oxidized. Thus, oxidization-induced release in the intracellular space might be possible.

## 4. Conclusions

Oxidation- and temperature-responsive polymeric micelles were prepared using an oxidizable amphiphilic LCST copolymer (i.e., P(HEA-*co*-PVS)(97/3)) for the delivery of DOX. The interfacial activity and LCST of P(HEA-*co*-PVS) were markedly affected by treatment with H_2_O_2_, possibly because PVS would be oxidized and become hydrophilic and the amphiphilicity of the copolymer would decrease. The release of the dye loaded in the copolymer micelle took place more extensively when the H_2_O_2_ concentration was higher, possibly because the oxidation and the solubilization of the micelle would be more favorable at the higher concentration. DOX loaded in the micelles suppressed the in vitro growth of KB cells much more effectively than DOX loaded in a control micelle (i.e., P(HEA-*co*-ABZ) micelle) and free DOX, possibly because the micelle was taken up by KB cells, the LCST of the copolymer would increase, and demicellization would be able to take place. In fact, FACS and CLSM revealed that DOX-loaded micelles were more readily internalized into KB cells. The oxidation- and temperature-responsive polymeric micelle developed in the present study could be used as a drug carrier for the efficient delivery of DOX to enhance its anticancer efficacy.

## Figures and Tables

**Table 1:** The molecular weight of copolymers.

Sample	M_w_	M_n_	PDI
P(HEA-*co*-PVS)(100/0)	12,674	10,522	1.20
P(HEA-*co*-PVS)(99/1)	11,460	9764	1.17
P(HEA-*co*-PVS)(98/2)	11,843	9947	1.19
P(HEA-*co*-PVS)(97/3)	11,952	10,042	1.19
P(HEA-*co*-ABZ)	11,799	9901	1.19

**Table 2:** The half maximal inhibitory concentration (IC_50_) values of free doxorubicin (DOX), P(HEA-*co*-PVS)(97/3)/DOX micelle, and P(HEA-*co*-ABZ)/DOX micelle for KB cells.

Sample	IC_50_ (μg/mL)
Free DOX	2.3
P(HEA-*co*-PVS)(97/3)/DOX micelle	1.62
P(HEA-*co*-ABZ)/DOX micelle	2.02
